# Nestin overexpression in hepatocellular carcinoma associates with epithelial-mesenchymal transition and chemoresistance

**DOI:** 10.1186/s13046-016-0387-y

**Published:** 2016-07-13

**Authors:** Yan Zhang, Shan Zeng, Junli Ma, Ganlu Deng, Yanlin Qu, Cao Guo, Hong Shen

**Affiliations:** Department of Oncology, Xiangya Hospital, Central South University, Changsha, Hunan 410008 China; Institute of Medical Sciences, Xiangya Hospital, Central South University, Changsha, Hunan 410008 China; Key Laboratory for Molecular Radiation Oncology of Hunan Province, Xiangya Hospital, Central South University, Changsha, Hunan 410008 China

**Keywords:** HCC, Nestin, EMT, Chemoresistance, Prognosis

## Abstract

**Background:**

Nestin expression has been reported to be associated with the prognosis of many solid tumors including human hepatocellular carcinoma (HCC). The present study aimed to identify the role, if any, of Nestin in the chemotherapeutic treatment of HCC.

**Methods:**

We determined Nestin expression in nine HCC cell lines and 220 tissue samples of advanced HCC patients (retrospectively registered) treated with FOLFOX regimens. We examined the correlations between Nestin expression and clinicopatholgical variables and HCC prognosis. Also, we used in vitro and in vivo methods to determine the effects of Nestin expression on HCC cell invasion, migration and chemosensitivity.

**Results:**

Nestin expression was significantly increased in HCC tissues and drug-resistant cell lines, and the presence of high levels of Nestin was associated with poor survival. We also showed that drug-resistance occurred in HCC cells with epithelial-mesenchymal transition (EMT), which in turn enhanced invasion ability. Nestin depletion reversed drug-resistance in the Bel-7402/5-FU and Bel-7402/ADM cell lines. Nestin knockdown enhanced chemotherapeutic efficacy in nude mice. Moreover, Nestin up-regulation in Bel-7402 was associated with the activation of Wnt/β-catenin signaling.

**Conclusion:**

Our findings suggest that Nestin inhibitors may be useful for the chemotherapy of HCC.

**Electronic supplementary material:**

The online version of this article (doi:10.1186/s13046-016-0387-y) contains supplementary material, which is available to authorized users.

## Background

Human hepatocellular carcinoma (HCC) is the fifth most prevalent cancer and the fourth most common cause of cancer-related death worldwide [1]. In 2008, 55 % of all new HCC cases reported globally were diagnosed in China [2], with a majority of HCC patients ineligible for curative operation due to advanced stage or metastasis [2, 3]. Despite some advances in HCC treatment - chemotherapy regimens (e.g., Doxorubicin and FOLFOX) and multi-kinase inhibitor Sorafenib - cancer mortality rates remain high [1, 3, 4]. An important factor causing ineffective chemotherapy is drug-resistance, which is complicated by the heterogeneity and multiple etiologies of HCC [5, 6]. The mechanisms responsible for multiple drug resistance (MDR) in HCC remain poorly understood.

Evidence suggests that the epithelial-mesenchymal transition (EMT) (characterized by downregulation of epithelial markers such as E-cadherin and ZO-1, and upregulation of mesenchymal markers such as Fibronectin and Vimentin) is a crucial event in tumor invasion and metastasis [7]. EMT is prompted by intracellular events including genetic and epigenetic changes, as well as signals from the tumor microenvironment e.g., TGF-β, Wnt/β-catenin, NF-kB, Notch, and RTK/Ras signaling. Wnt/β-catenin signaling is critical for promoting self-renewal, tumorigenicity and drug-resistance properties of HCC [8, 9]. Nestin, a type VI intermediate filament protein, was first identified as a neural stem cell marker [10]; subsequent reports suggested that Nestin expression indicates poor prognosis in many types of solid human tumors [11], including HCC [12, 13]. Nestin regulates prostate cancer cell invasion [14], EMT-related factors in pancreatic adenocarcinoma [15], and drug resistance in other cancers [16, 17]. However, whether Nestin is involved in regulating the drug-resistance of HCC remains unknown.

We hypothesize that elevated Nestin expression promotes the development of human HCC, reduces HCC chemo-sensitivity, and is associated with poor therapeutic outcomes. Here we measured Nestin expression in HCC tissues and determined the association between Nestin and human HCC prognosis. Moreover, we investigated the involvement of Nestin in HCC cell invasion, migration and chemo-resistance using in vitro and in vivo models.

## Methods

### Patients and tissue specimens

From January 2005 to July 2012, we randomly collected tumor and matched adjacent non-tumor tissue specimens from 220 advanced HCC patients (retrospectively registered). For each cancer sample, diagnoses were made by two independent pathologists. We used patient inclusion criteria similar to that in a previous study [18]. All enrolled patients: (i) received curative operation at diagnosis and suffered recurrence after HCC operation, (ii) had received first-line FOLFOX chemotherapy (5-Fluorouracil combined with Oxaliplatin) after recurrence, (iii) had not received any other anti-tumor therapy before disease progression. Chemotherapy doses varied depending on toxicity and responsiveness. Clinical data was obtained from electronic medical records, and survival data was obtained from the Tumor Registry at Xiangya Hospital. The clinicopathological features of the HCC patients were summarized in Table 1. Response to treatment was evaluated according to the Response Evaluation Criteria in Solid Tumors (RECIST, Version 1.1) guidelines [19]. Both progression-free survival (PFS) and overall survival (OS) were calculated beginning from the date of initial chemotherapy treatment. PFS was measured to the date of first clinical progression or death from any cause. OS was measured to the date of death from any cause and patients alive at the last inquiry were excluded. The primary end point was PFS and the secondary end point was OS.

**Table 1:** Main clinical features according to Nestin status in patients with advance HCC (*n* = 220)

Clinical and pathological indexes			Nestin expression		
		Total	Low	High	*P*
		n	n (%)	n (%)	
Age (years)	≤ 60	118	52(44.1)	66(55.9)	0.119
	> 60	102	54(52.9)	48(47.1)	
Gender	Male	109	49(45.0)	60(55.0)	0.208
	Female	111	57(51.4)	54(48.6)	
HBsAg	Negative	120	59(49.2)	61(50.8)	0.427
	Positive	100	47(47.0)	53(53.0)	
Albumin	≤ 35 g/L	124	57(46.0)	67(54.0)	0.498
	> 35 g/L	96	49(51.0)	47(49.0)	
Liver cirrhosis	Absent	73	40(54.8)	33(45.2)	0.107
	Present	147	66(44.9)	81(55.1)	
Child-Pugh stage	A	120	57(47.5)	63(52.5)	0.466
	B	100	49(49.0)	51(51.0)	
AFP (ng/ml)	≤ 20	77	35(45.5)	42(54.5)	0.326
	> 20	143	71(49.7)	72(50.3)	
Edmondson-Steiner grade	I-II	96	52(54.2)	44(45.8)	0.077
	III-IV	124	54(43.5)	70(56.5)	
Macroscopic vascular invasion	Absent	104	61(58.7)	43(41.3)	**0.002** ^*^
	Present	116	45(38.8)	71(61.2)	
BCLC stage	B	86	52(60.5)	34(39.5)	**0.003** ^*^
	C	134	54(40.3)	80(59.7)	
Disease status	Confined to the liver	100	57(57.0)	43(43.0)	**0.012** ^*^
	Metastatic disease	120	49(40.8)	71(59.2)	

*Abbreviations*: *BCLC stage* Barcelona Clinic Liver Cancer

^*^significant difference is shown in bold

### IHC and grading of Nestin expression levels

Nestin protein expression in formaldehyde-fixed, deparaffinized tissue sections was assessed by the standard automated IHC procedure after microwave-enhanced epitope retrieval. The sections were incubated with either monoclonal anti-Nestin antibody at a dilution of 1:200, or phosphate-buffered saline as the negative control. The intensity of anti-Nestin staining was scored semi-quantitatively as described previously [20]. Cells with yellow to brown cytoplasmic staining were considered to be Nestin-positive (+) cells. Nestin expression levels in individual tumor tissues were assigned a score based on the following criteria: 0 if < 1 % of neoplastic cells were Nestin+; 1+, if neoplastic cells were 1 to 10 % Nestin+; and 2+ if ≥ 10 % of neoplastic cells were Nestin+. Individual sections with 1+ or 2+ anti-Nestin staining were considered positive tissue specimens.

### Cell lines and cell culture

Human HCC cell lines — SMMC-7721, HepG2, Huh7, Hep3B, MHCC97H, HCCLM3 and Bel-7402 — were purchased from Shanghai Institute of Biochemistry and Cell Biology, Chinese Academy of Science. Drug resistant cell lines — Bel-7402/5-Fludrouracil (5-FU) and Bel-7402/Adriamycin (ADM) — were obtained by exposing Bel-7402 to stepwise increases in 5-FU or ADM according to reported methods [21, 22].

### Transfection

We used lentivirus containing shRNA-Nestin, prepared as previously described [23], and expression vectors containing the full-length Nestin cDNA fragment (Invitrogen, Carlsbad, CA). The presence of the inserts was verified by sequence analysis (GeneChem, Shanghai, China). Down-regulated expression or over-expression of Nestin was confirmed by qRT-PCR and Western blot. All experiments were performed in triplicate.

### Chemosensitivity detection

The half-maximal inhibitory concentration (IC_50_) values of 5-FU, Oxaliplatin (L-OHP) and ADM were calculated for each cell line. HCC cells were diluted to a density of 8 × 10^3^ cells/200 μL before being seeded in 96-well plates and incubated at 37 °C for 24 h. All anti-cancer drugs were freshly prepared. Cell survival rates and dose-dependent curves of drugs were determined using MTT assays, IC50 values were calculated and analyzed by SPSS software (Version 13.0, Chicago, IL).

### Quantitative real-time RT-PCR

mRNA expression was quantitatively analyzed using the SYBR Green fluorescent-based assay (TaKaRa Bio Inc., Otsu, Japan). The primers for real-time PCR are listed in Additional file 1: Table S1. The relative mRNA expression levels of target genes were normalized to the internal control of GAPDH.

### Western blotting

Total protein or nuclear protein was separated by SDS-PAGE under reducing conditions and then transferred onto PVDF membrane (Millipore, Bedford, MA). The blotted membranes were blocked in 5 mg/mL skim milk and then incubated with the primary antibodies. HRP-conjugated IgG was used as the secondary antibody. GAPDH and Histone protein were used as the internal controls for total protein and nuclear protein respectively. Imaging of western blots was visualized with the ChemiDoc XRS+ system, and the quantitative analysis was performed with Image Lab software (Bio-Rad, Hercules, CA).

### In vitro wound healing and transwell invasion assays

HCC cells were seeded into 6-well plates and cultured for 1 day. After the cells achieved near 100 % confluence, a scraped line was created with a 200 ul pipette tip. We then replaced the medium and cultured the cells with serum-free medium for 24 h. The speed of wound closure was imaged with an inverted microscope (Nikon Eclipse TE2000-S, Tokyo, Japan) and the rate of wound closure was calculated. The transwell chambers used for the invasion assay contained polycarbonate filters with 8-μm pore size (BD Biosciences, San Jose, CA) whose upper surfaces were coated with a BD Growth Factor-Reduced Matrigel Matrix. Medium containing 10 % fetal bovine serum was placed in the lower chambers to act as a chemo-attractant. Cells (2 × 10^4^ in 500 μl serum-free medium) were placed in the upper chamber and incubated at 37 °C for 24 h. The cells that penetrated the Matrigel-coated filter were first stained with 0.1 % crystal violet hydrate solution, and then counted in 15 randomly selected fields; the mean number of cells per field was recorded. Each assay was performed on triplicate filters, and each experiment was repeated three times.

### Immunofluorescence staining

HCC cells were grown in glass-bottom dishes overnight, fixed in ice-cold 4 % paraformaldehyde for 30 min, permeabilized with 0.02 % Triton X-100 for 10 min, and blocked with 1 % Bull Serum Albumin (BSA) for 1 h at room temperature. Cells were then incubated overnight at 4 °C with anti-E-Cadherin, and anti-Vimentin primary antibodies followed by either Alexa 488 or 546-conjugated secondary antibody and the DNA dye DAPI for 3 min at room temperature. Immunofluorescence images were acquired using an upright microscope (Leica DM 5000B, German) at 405, 488, or 546 nm, which was controlled by corresponding fluorescence filter block.

### Primary HCC mouse model

For in vivo studies, 4 week old male nu/nu mice were subcutaneously (s.c.) injected with 1× 10^7^ HCC cells in the left upper regions. Five weeks later, the subcutaneous tumors were removed and cut into pieces of the same size of 1 mm x 1 mm x 1 mm. These sterile pieces were immediately implanted into the liver under the capsula fibrosa in a separate group of 6 week old male mice in order to mimic primary HCC. To explore the effects of altered Nestin expression on chemotherapeutic efficacy, we selected the classical antitumor drug 5-FU. Four weeks after orthotopic implantation, the mice were treated daily for a period of 2 weeks with either DMSO as a vehicle control or 5-FU (10 mg/kg) by intraperitoneal administration [24]. Subsequently, animals received intravenous injections of MMPSense™ 750 FAST fluorescent imaging agent (2 nmol agent formulated in 1X PBS); 6 h later, mice were imaged by Fluorescence Molecular Tomography (FMT-4000, PerkinElmer, Boston, MA). Tumor Size was taken as the Fluorescence Volume as calculated by the Quantitative Tomography In Vivo Imaging software of TrueQuant™ (PerkinElmer).

### Statistics

Data were analyzed using SPSS software (Version 13.0). *χ*2 tests were used for comparisons between Nestin expression and the clinicopatholgical characteristics of HCC. Survival curves were calculated using the Kaplan-Meier method and compared by the log-rank test. Cox proportional hazards regression modeling was performed to identify the factors independently associated with survival. A *P*-value < 0.05 (two-sided) was considered statistically significant.

## Results

### Relationship between Nestin expression and the clinical characteristics of HCC

**Fig. 1:**
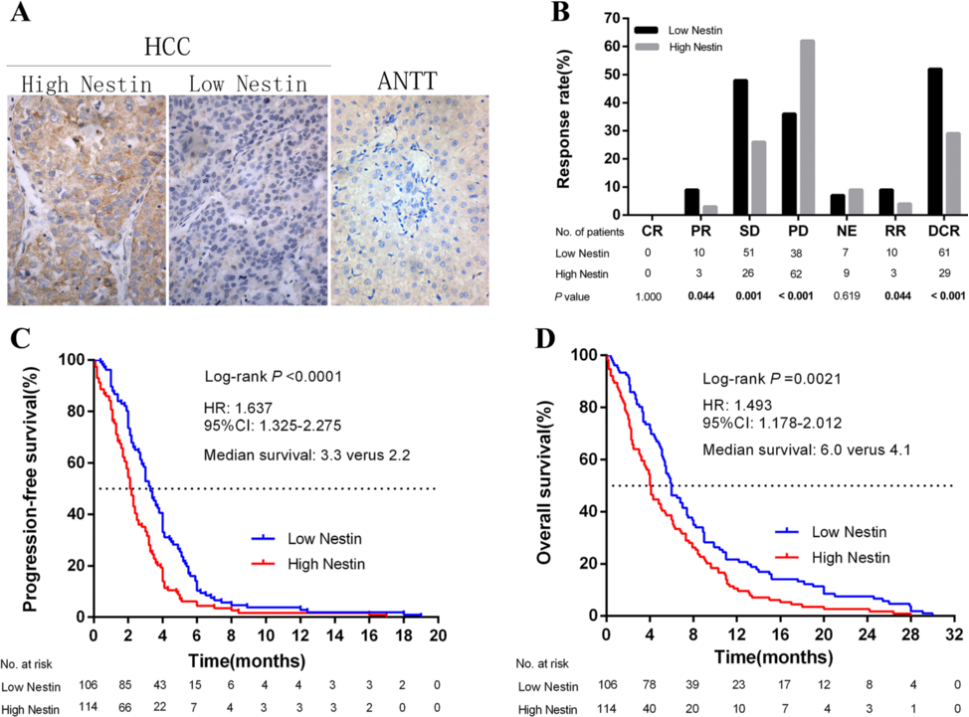
Increased expression of Nestin in HCC tissue. **a** Representative immunohistochemical staining of Nestin protein in HCC tissues and adjacent non-tumor tissue (ANTT) (Original magnification: × 400). **b** The association between Nestin expression and chemotherapeutic response (FOLFOX regimens) was analyzed. More patients in low Nestin group achieved PR, SD, RR and DCR, and less PD, all *P* < 0.05. HCC patients with higher Nestin expression have poorer PFS (**c**) and OS (**d**) than those with lower Nestin expression. CR, PR, SD, PD, NE, RR and DCR represent complete response, partial response, stable disease, progressive disease, not evaluated, response rate (RR) and disease control rate (DCR) respectively. RR = CR + PR, DCR = CR + PR + SD

We determined the expression levels of Nestin protien in HCC tumor and adjacent non-tumor tissue IHC. Nestin protein expression was mainly observed in cytoplasm. Nestin expression was significantly higher in tumor tissues as compared to adjacent non-tumor tissue (Fig. 1a). Table 1 shows the relationships between Nestin expression and clinical variables. A high level of Nestin expression was significantly associated with serous macroscopic vascular invasion (*P* = 0.002), advanced BCLC stage (*P* < 0.003) and metastatic disease (*P* = 0.012). No correlations were found between Nestin expression level and the other clinicopatholgical variables considered (all *P* > 0.05).

### Association of Nestin with chemotherapeutic response and survival

All patients suffering HCC recurrence received systemic chemotherapy: first-line FOLFOX regimens included L-OHP, Leucovorin (LV), and 5-FU. The median follow-up time for all participants was 17.3 months (range, 1 to 39 months). Of all 220 patients, 10 (4.5 %) patients failed to follow-up, while 210 (95.5 %) patients developed progressive disease or died. Figure 1b shows the association between Nestin expression and chemotherapeutic efficacy. No patient achieved a complete response (CR) in either the low or high Nestin groups. Compared with the high Nestin group, there was a greater number of patients in the low Nestin group who achieved a partial response (PR), stable disease (SD), disease control rate (DCR) and response rate (RR); while fewer patients in the low Nestin group suffered progressive disease (PD), all *P* < 0.05.

### Nestin expression correlates with tumor progression and overall survival

Nestin-high patients exhibited significantly shorter PFS as compared to Nestin-low patients (Fig. 1c, *P* < 0.0001). Also, Nestin-high patients had significantly shorter OS time compared to Nestin-low patients (Fig. 1d, *P* = 0.0021). A multivariable Cox proportional hazards model was constructed to assess the independent predictive value of Nestin as a prognostic indicator for HCC. This analysis indicated that advanced BCLC stage (HR = 1.892, *P* < 0.001) and high Nestin expression (HR = 1.973; *P* < 0.001) were independent predictive indicators of PFS, while advanced BCLC stage (HR = 1.811, *P* < 0.001), high Nestin expression (HR = 2.375; *P* < 0.001) and metastatic disease (HR = 1.557; *P* = 0.010) were found to be independent prognostic indicators of OS in HCC patients (Table 2).

**Table 2:** Variables associated with advanced HCC analyzed by a multivariate Cox proportional hazards regression model

Clinical and pathological variables	PFS			OS		
	HR	95 % CI	*P*	HR	95 % CI	*P*
Age, years						
(≤ 65 versus > 65)	1.024	0.768–1.366	0.871	1.082	0.818–1.432	0.580
Gender						
(Male versus Female)	0.877	0.664–1.158	0.356	0.920	0.629–1.224	0.568
HBsAg						
(Negative versus Positive)	0.961	0.725–1.247	0.781	1.002	0.758–1.325	0.990
Albumin						
(≤ 35 g/L versus > 35 g/L)	0.974	0.723–1.310	0.859	0.933	0.690–1.262	0.653
Liver cirrhosis						
(Absence versus Presence)	1.163	0.859–1.574	0.329	0.945	0.701–1.274	0.710
Child-Pugh classification						
(A versus B)	0.938	0.701–1.255	0.666	0.946	0.710–1.259	0.702
AFP, ng/ml						
(≤ 20 versus > 20)	0.994	0.723–1.367	0.972	0.824	0.602–1.127	0.227
Edmondson-Steiner grade						
(I/II versus III/IV)	0.894	0.675–1.183	0.433	1.314	0.985–1.755	0.064
Macroscopic vascular invasion						
(Absence versus Presence)	1.221	0.885–1.683	0.225	2.023	1.465–2.795	**< 0.001** ^*^
BCLC stage						
(B versus C)	1.892	1.369–2.614	**< 0.001** ^*^	1.811	1.340–2.447	**< 0.001** ^*^
Disease status						
(Confined to the liver versus Metastatic disease)	1.118	0.803–1.557	0.508	1.557	1.114–2.176	**0.010** ^*^
Nestin expression level						
(Low versus High)	1.973	1.433–2.716	**< 0.001** ^*^	2.375	1.744–3.232	**< 0.001** ^*^

*Abbreviations*: *CI* confidence interval, *HR* hazard ratio

^*^Significant difference is shown in bold

### Nestin expression level is upregulated in chemoresistant HCC sublines

**Fig. 2:**
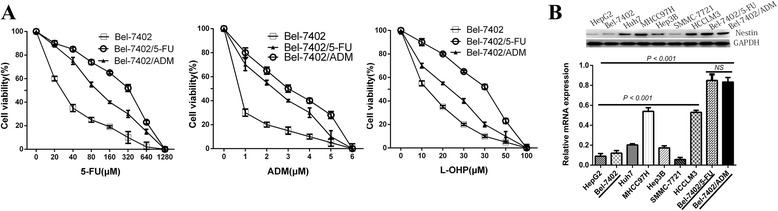
The expression of Nestin and the different chemo-sensitivities in various HCC cell lines. **a** The IC_50_ values of the parental (Bel-7402) and chemo-resistant cell lines (Bel-7402/5-FU and Bel-7402/ADM) were determined by MTT assays. **b** In all nine examined HCC cells lines, quantitative reverse transcription-polymerase chain reaction (qRT-PCR) and Western blotting demonstrated the high Nestin mRNA and protein expression in two highly invasive HCC cell lines (MHCC97H and HCCLM3) and in the two drug-resistant sublines (Bel-7402/5-FU and Bel-7402/ADM)

To explore the possible mechanisms of chemoresistance in HCC, two HCC sublines resistant to 5-FU or ADM were established. As shown in Fig. 2a, the HCC cell lines Bel-7402/5-FU and Bel-7402/ADM were more resistant to chemotherapeutic drugs than parental cells (The IC_50_s of anticancer drugs for HCC cells were showed in Additional file 2: Table S2). Two highly invasive HCC sub-lines (MHCC97H, HCCLM3) and two chemoresistant sublines (Bel-7402/5-FU, Bel-7402/ADM) showed high mRNA and protein levels of Nestin (Fig. 2b).

### Chemoresistant HCC sublines have an EMT phenotype and high invasion ability

**Fig. 3:**
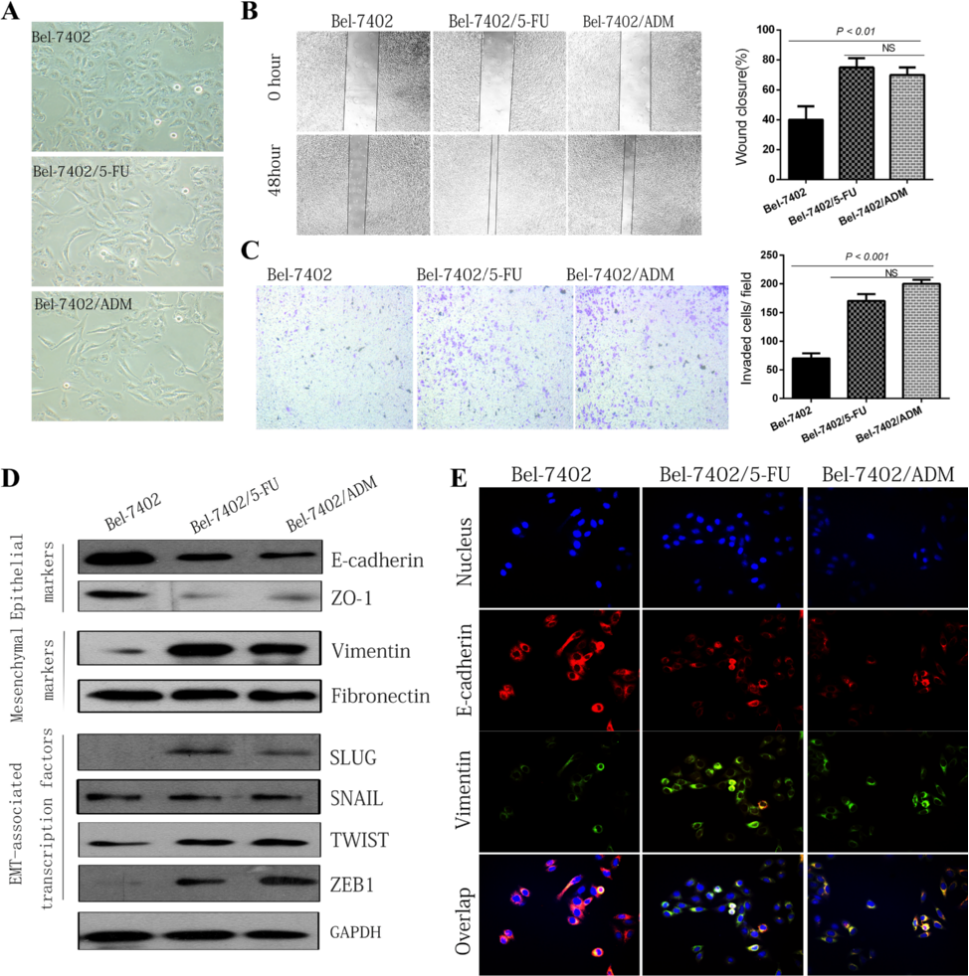
Chemoresistant HCC sublines exhibit characteristics of epithelial-mesenchymal transition (EMT). **a** Cellular morphology of Bel-7402/5-FU and Bel-7402/ADM was significantly changed compared with Bel-7402. The wound-healing assay (**b**) and transwell assay (**c**) were performed to analyze the difference in motility and invasion between parental (Bel-7402) and drug-resistant cell lines (Bel-7402/5-FU and Bel-7402/ADM). The percentage of wound healing for cells passing through the transwell membranes of each well was calculated and is compared in the diagrams. **d** Western blotting assays showed the expression of EMT-related proteins in parental (Bel-7402) and drug-resistant cell lines (Bel-7402/5-FU and Bel-7402/ADM). **e** Immunofluorescence staining of nuclei, E-Cadherin and Vimentin. Images were taken using a fluorescense microscope under excitation at 405 nm, 488 nm, or 546 nm (Scale bars: 100 μm)

Compared with parental cells, the chemoresistant HCC sublines exhibited a mesenchymal-like morphology and fewer cell-cell junctions (Fig. 3a). In vitro wound healing and transwell invasion assays showed that HCC sublines were more invasive than parental cells (Fig. 3b and c). Additional file 3: Figure S1 shows the mRNA expression levels of EMT markers in different groups of Bel-7402 cells measured by quantitative real-time RT-PCR. The protein levels of the mesenchymal marker Vimentin and EMT-associated transcription factors SLUG and ZEB1 were upregulated in drug resistant cells compared with parental cells, whereas epithelial markers E-cadherin and ZO-1 were down-regulated (Fig. 3d). These differences in epithelial and mesenchymal marker (E-Cadherin and Vimentin) expression were verified by immunofluorescence staining (Fig. 3e).

### Downregulation of Nestin reverses drug resistance in HCC cell lines

**Fig. 4:**
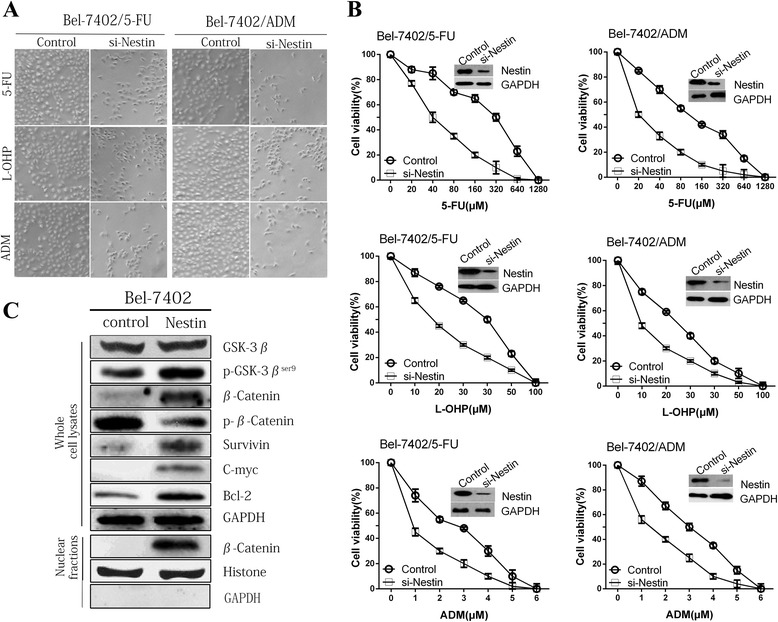
The signaling pathways involved in Nestin-regulated HCC multidrug resistance (MDR). **a** The cell morphology of parental (Bel-7402) and resistant cell lines (Bel-7402/5-FU and Bel-7402/ADM) transfected with si-Nestin was significantly changed by 5-FU, L-OHP and ADM treatment. **b** MTT assay demonstrated significantly decreased cell viability in Si-Nestin-transfected resistant cell lines (Bel-7402/5-FU and Bel-7402/ADM) compared to Si-Controls, following 5-FU, L-OHP and ADM treatments respectively (all *P* < 0.05). **c** The signaling pathways involved in Nestin-regulated MDR of HCC cells. As compared to the Bel-7402-control, levels of GSK-3β and p-β-Catenin were significantly lower, while p-GSK-3β^Ser9^, total β-catenin, survivin, C-myc, Bcl-2 and nuclear β-catenin levels were higher in Bel-7402-Nestin

The higher expression of Nestin in chemoresistant cells as compared to parental cells suggests that Nestin may play a causal role in chemoresistance. Therefore, we determined the effects of Nestin suppression on the MDR exhibited by HCC cells to 5-FU, L-OHP and ADM (i.e., common chemotherapy drugs administered to HCC patients). si-Nestin-Bel-7402/5-FU and si-Nestin-Bel-7402/ADM cells exhibited cell morphology changes in response to 5-FU, DDP and ADM treatment (Fig. 4a). The MTT assay demonstrated that, across a range of concentrations, anticancer drugs (5-FU, L-OHP and ADM) were more effective in the si-Nestin group as compared to the Control group (Fig. 4b, all *P* < 0.05). The IC_50_s of 5-FU, L-OHP and ADM in si-Nestin-Bel-7402/5-FU and si-Nestin-Bel-7402/ADM cells were significantly decreased, as compared with that of the control HCC cells (Additional file 4: Table S3).

### Nestin promotes β-catenin expression, activation, and nuclear localization in HCC cells

The role of Nestin in the mechanism of HCC chemoresistance has not yet been established; however, evidence suggests that Wnt/β-Catenin signaling may be involved. A hallmark of hyperactivation of Wnt/β-catenin signaling is degradation of the APC/Axin/glycogen synthase kinase (GSK)-3β complex and upregulation of GSK-3β phosphorylation (Ser9 site). Nestin upregulation in the Bel-7402-Nestin cell line resulted in increased activation of GSK-3β (p-GSK-3β^Ser9)^, increased total β-catenin and nuclear β-catenin expression (Fig. 4c), and increased mRNA levels of survivin, c-myc, and Bcl-2 (proteins encoded by β-catenin target genes; Additional file 5: Figure S2).

### Nestin Silencing inhibits EMT and increases chemosensitivity of Bel-7402/5-FU in vivo

**Fig. 5:**
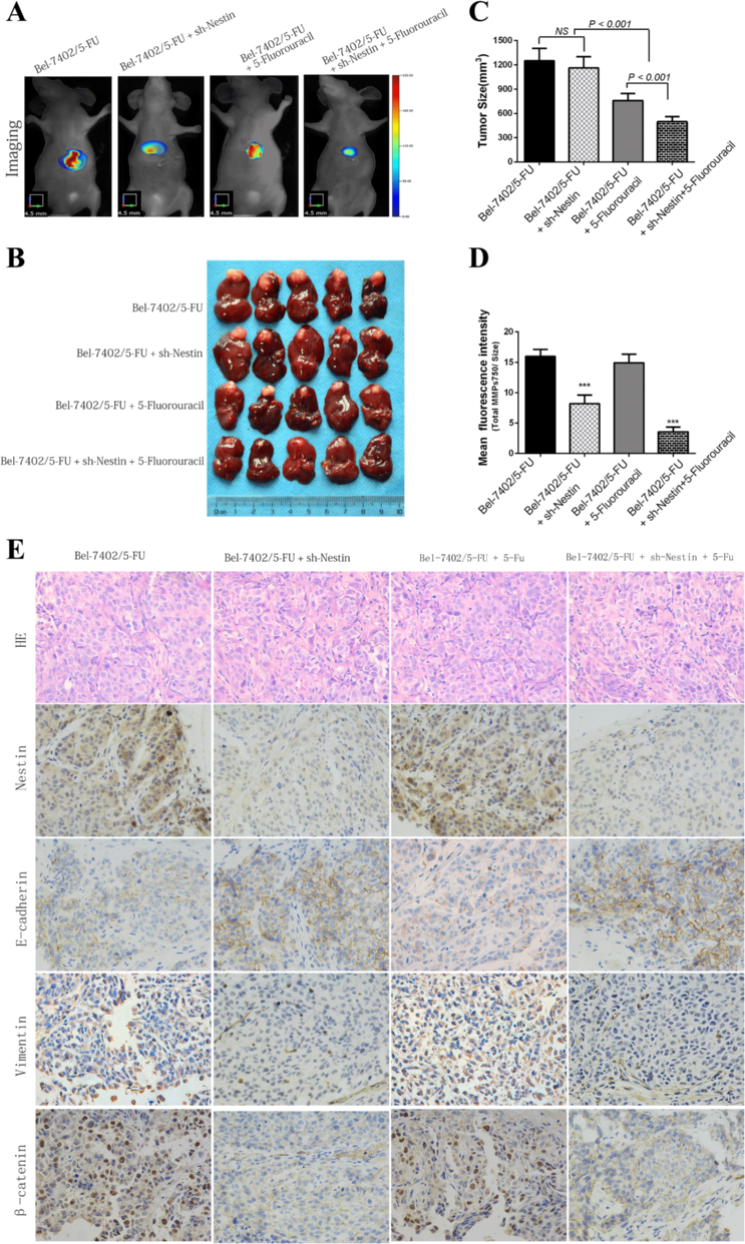
Silencing of Nestin on chemosensitivity *in vivo*. **a** The mice were imaged by Fluorescence Molecular Tomography (FMT-4000, PerkinElmer). **b** Images of primary tumors in Nestin silenced groups, corresponding controls groups, including intraperitoneal injection of 5-FU groups. **c** Primary tumor size was calculated by TrueQuant™ (the Quantitative Tomography In Vivo Imaging Software). The tumors in mice that received 5-FU treatment and stable Nestin-shRNA transfection were smaller than those either treated with 5-FU or transfection alone (all *P* < 0.001). **d** The mean fluorescence intensity (Total MMPs/Tumor Size) of tumors in Si-Nestin group were smaller than those in Si-Control group (*P* < 0.001). **e** Representative HE and IHC staining of proteins (Nestin, E-cadherin, Vimentin and β-Catenin) in tumor tissues of nude mice. (Original magnification: ×400)

Tumors in mice that received 5-FU plus stable si-Nestin transfection were smaller than those treated either with 5-FU or si-Nestin transfection alone (all *P* < 0.001, Fig. 5a, b and c). The mean intensity of fluorescence (Total MMPs/Tumor Size), Vimentin and β-catenin (especially in nuclear) decreased, while E-cadherin increased significantly in the Si-Nestin treated group as compared to the Si-Control group (Fig. 5d and e).

## Discussion

Nestin is a class VI intermediate filament protein that is overexpressed in certain cancers. It has been reported to regulate tumor growth, migration, invasion, angiogenesis, metastasis, apoptosis, DNA damage repair, and tissue regeneration in different types of cancer cells [11, 14, 20, 25]. Nestin is frequently up-regulated in HCC, and has been previously shown to be significantly correlated with tumor angiogenesis and poor prognosis in HCC patients [12]. A recent analysis of 89 HCC patients by the Memorial Sloan Kettering Cancer Center indicated that Nestin overexpression may be a key, early event driving the growth of HCC [13]. Here we showed that Nestin expression is significantly elevated in HCC tissues, which is consistent with the previous study [26].

Our results indicate that Nestin is a candidate promoter of EMT, chemo-resistance and poor outcome in HCC patients. Patients with high Nestin expression exhibited low disease control rate with FOLFOX treatment. More specifically we showed that chemo-resistant HCC cell lines having increased Nestin expression exhibited a typical profile of molecular changes: up-regulation of mesenchymal markers, EMT-associated transcription factors (Slug and ZEB1), and the down-regulation of epithelial markers. Nestin knockdown in two drug-resistant cell lines consistently increased chemo-sensitivity. Consistent with this finding, we showed Nestin involvement in chemo-resistance in vivo using xenograft HCC tumors in nude mice treated with 5-FU: Nestin depletion resulted in repression of EMT and enhanced chemo-sensitivity. EMT-associated transcription factors are commonly activated in aggressive HCC. Previous IHC analysis of human HCC specimens showed a down-regulation of E-cadherin and dissociation of β-catenin from cell borders (particularly at the tumor invasive front) which also correlated with Snail and Slug expression [27]. Expression of Twist, a negative regulator of E-cadherin transcription, is correlated with HCC metastasis and is associated with EMT changes and HCC cell invasiveness [28, 29]. In the present study, we found that chemo-resistant HCC cell lines with increased Nestin expression exhibited up-regulation of Slug and ZEB1, but no remarkable change in Snail or Twist expression. Another study has demonstrated an association between Slug expression and EMT, cancer stem cell (CSCs) phenotype and angiogenesis in HCC patients [30]. Moreover, positive ZEB-1 expression and loss of E-cadherin expression are correlated with poor prognosis in HCC patients. Also, the malignancy of ZEB-1 positive tumors involves EMT-related factors [31]. EMT, as well as tumor migration and invasion of HCC cells is promoted by the oncogene HOXD9; while ZEB1 knockdown inhibits these HOXD9-induced effects [32]. Our results are in consistent with these data. Still, it’s worth noting that the xenograft derived from Bel-7402/5-FU treated with 5-FU exhibited significantly smaller-sized tumor than Bel-7402/5-FU with no 5-FU intervention. There is a possibility that during tumor formation, epigenetic regulation has been changed and the susceptibility to 5-FU is again recovered. Altogether, we observed that chemoresistance existed in HCC cells with EMT, thereby enhancing tumor invasion and metastasis; this may provide an explanation for the poor prognostic outcomes in chemo-treated HCC patients with Nestin-overexpressed tumors.

The present study reveals the role of Nestin in molecular mechanisms of drug-resistance exhibited by HCC cells. The up-regulation of bcl-2 and survivin expression may explain, at least in part, the chemoresistance induced by Nestin overexpression. These results are consistent with recent findings showing that the expression of P-gp is associated with expression of Nestin in human acute myeloid leukemia cell lines (MOLM-13 and SKM-1) [33]. However, the role of Nestin in the regulation of drug transport remains to be elucidated. Previous research suggests that p53 mutations contribute to arsenic trioxide resistance and enhanced metastatic potential of HCC cells [34]. Tschaharganeh, DF et al. demonstrated that Nestin functionally contributes to p53 inactivation to promote liver tumorigenesis, and the inactivation of p53 prompts mature hepatocytes to dedifferentiate into Nestin-positive progenitor-like cells, which subsequently differentiate into HCC [13]. Intra-tumoral heterogeneity makes the elimination of cancer cells challenging, since the responsiveness to chemotherapy can vary [35]. Metabolic reprogramming is required for both malignant transformation and tumor development, including invasion, metastasis and chemotherapy resistance [36, 37]. Also, p53 is demonstrated to play a pivotal role in EMT and metastasis of HCC cells [38]. Therefore, we suggest that the inactivation of p53 is a probable mechanism through which Nestin promotes drug-resistance and EMT in HCC cell lines. On the other hand, hepatic cancer cells having high Nestin expression tend to undergo EMT, where the splicing form of CD44 is changed from CD44 variant-dominant to CD44 standard-dominant status [39, 40]. This is consistent with a previous report that CD44v and c-Myc expression tend to be inversely correlated, especially at the invasive front of tumorous tissue specimens [41].

The Wnt/β-Catenin signaling pathway is implicated in numerous aspects of development, cell biology and physiology including the EMT process and chemo-resistance [42]. Activation of Wnt/β-Catenin signaling is reportedly involved in P-gp-dependent and independent MDR [43]. Increasing evidence suggests that the development of chemotherapy resistance is likely to correlate with changes in EMT phenotype [44]. For instance, low E-cadherin expression and increased mesenchymal phenotype can lead to apoptosis resistance. This is supported by our observation that Nestin-overexpressing cells display a mesenchymal-like phenotype (E-cadherin-negative and N-cadherin-positive). In this study, we observed that the chemoresistant HCC cell lines with increased Nestin expression had different epithelial cell morphology compared to that of parent cells. The correlation between Nestin and EMT suggests possible involvement of Nestin in drug-resistance and tumor invasion. Nestin could induce MDR in HCC cells though the phosphorylation and inactivation of GSK-3β, as well as though enhanced expression, activation, and nuclear localization of β-catenin. Chemoresistance may be induced after exposure to 5-FU, ADM and L-OHP via the Nestin and Wnt/β-catenin signaling pathway. Nestin and Wnt/β-catenin signaling may exert multiple functions related to drug resistance, EMT, and migration/invasion of HCC cells. CD44, one of the main Wnt/beta-catenin signaling target molecules as well as cancer stem cell marker molecules [39, 40], is involved in acquisition of resistance to oxidative stress induced by the action of chemotherapeutic agents on the xCT transporter [40, 41]. Li-Fraumeni syndrome (LFS), a hereditary syndrome characterized by predisposition to cancer that is commonly associated with a germline mutation in the tumor suppressor gene p53 [45]. In a case report about LFS, surviving tumor cells population in the minimal residual disease state post chemotherapy were found to be enriched in CD44 variant 8-10-positive cancer stem-like cells [46]. Given our finding that high levels of Nestin expression induce EMT, there is a high likelihood that Nestin+/CD44v8-10+ cells correspond to those cancer stem cells that give rise to relapse, metastasis and chemo-resistance. Tumor tissue consists of a diverse cellular population that are heterogeneous in terms of their dependency on the Warburg effect and their mitochondrial metabolism. Mitochondria play an important role in cancer metabolism, particularily in terms of their involvement in glutaminolysis [36, 37, 47]. Importantly, some evidence suggests that oncogenic c-Myc mediates elevation of glutaminolysis in cancer cells [47–49]. Tumor heterogeneity is the result of the hierarchical model with cancer stem cells and non-cancer stem cells. Normal hepatic tissue is composed of oxidative hepatic cells located near the portal vein and hypoxic hepatic cells located near to the central vein. It is widely recognized that these cell groups exhibit metabolic symbiosis that varies according to their local nutrient and oxygen microenvironment [37]. Likewise, this is also the case in HCC tissues. It has been reported that the inflammatory cytokines produced by non-cancer stem cells due to chemotherapy leads to iatrogenic activation of cancer stem cells [36]. Metabolic reprogramming may also underlie the difficulty in treating HCC associated with high Nestin expression.

## Conclusion

In the present study, we clarified the relationship between Nestin overexpression and poor prognosis in recurrent HCC patients receiving FOLFOX regimen, and its association with chemoresistance and EMT in HCC cell lines. In terms of drug sensitivity and tumor recurrence, individualized therapies could be implemented to improve long-term survival. However, Nestin-mediated HCC chemoresistance and metastasis are needed to fully understand.

## Additional files

Additional file 1: Table S1. The primers sequences of qRT-PCR. (DOCX 14 kb) Additional file 2: Table S2. IC50s (μM/L) of anticancer drugs for HCC cells. (DOCX 14 kb) Additional file 3: Table S3. The influence of Nestin on IC50s (μM/L) of anticancer drugs for HCC cells. (TIF 304 kb) Additional file 4: Figure S1. The mRNA expression levels of EMT markers in different groups of Bel-7402 cells measured by quantitative real-time RT-PCR. (DOCX 13 kb) Additional file 5: Figure S2. Nestin upregulation in the Bel-7402-Nestin cell line resulted in increased mRNA levels of survivin, c-myc, and Bcl-2 (proteins encoded by β-catenin target genes). (TIF 128 kb)

## Abbreviations

ADM: adriamycin
AFP: alpha-fetoprotein
ANLT: adjacent non-tumorous liver tissue
EMT: epithelial-mesenchymal transition
5-FU: 5-Fludrouracil
GAPDH: glyseraldehyde-3-phosphate dehydrogenase
HCC: hepatocellular carcinoma
IHC: immunohistochemistry
L-OHP: oxaliplatin
LV: leucovorin, MMP, matrix metalloproteinase
MDR: multiple drug resistance
OS: overall survival
PFS: progression-free survival
siRNA: short interfering RNA
TNM: tumor-node-metastasis
