# Supplementary material for: Nestin overexpression in hepatocellular carcinoma associates with epithelial-mesenchymal transition and chemoresistance
Source: J Exp Clin Cancer Res. 2016 Jul 13;35:111. doi: 10.1186/s13046-016-0387-y (PMC4944516; doi:10.1186/s13046-016-0387-y)
Supplement: Additional file 1: Table S1. — The primers sequences of qRT-PCR. (DOCX 14 kb) [file 13046_2016_387_MOESM1_ESM.docx]

**Supplementary Table 1： The sequences of primers**

| Genes | primers |
| --- | --- |
| Nestin | F:5’-CTCCAAGAATGGAGGCTGTAGGAA -3’ |
|  | R:5’-CCTATGAGATGGAGCAGGCAAGA -3’ |
| E-cadherin | F:5’-TACGCCTGGGACTCCACCTA-3’ |
|  | R:5’-CCAGAAACGGAGGCCTGAT-3’ |
| ZO-1 | F:5’-CCATCTTTGGACCGATTGCTG -3’ |
|  | R:5’-TA ATGCCCGAGCTCCGATG-3’ |
| Vimentin | F:5’-ATGTGGATGTTTCCAAGCCTGAC -3’ |
|  | R:5’-GAGTGGGTATCAACCAGAGGGAG -3’ |
| Fibtonectin | F:5’-ATGGTGGTCTCAGTAGCA -3’ |
|  | R:5’-CTCATC-CGTGGTTGTATCA -3’ |
| α-SMA | F:5’-TGGTATTGTGCTGGACTCTG -3’ |
|  | R:5’-CCATCAGGCAGTTCGTAG -3’ |
| Snail | F:5’-CACTATGCCGCGCTCTTTC -3’ |
|  | R:5’-GCTGGAAGGTAAACTCl’GGATTAGA -3’ |
| Slug | F:5’-CTTTTTCTTGCCCTCACTGC -3’ |
|  | R:5’-ACAGCAGCCAGATTCCTCAT-3’ |
| Twist | F:5’-GGAGTCCGCAGTCTTACGAG -3’ |
|  | R:5’-TCTGGAGGACCTGGTAGAGG -3’ |
| Zeb-1 | F:5’-GAGAAGCGGAAGAACGTGAC -3’ |
|  | R:5’-GCTTGACTTTCAGCCCTGTC -3’ |
| Survivin | F:5’-AGAACTGGCCCTTCTTGGAGG -3’ |
|  | R:5’-CTTTTTATGTTCCTCTATGGGGTC-3’ |
| c-myc | F:5’-CGGATTCTCTGCTCTCCTCG -3’ |
|  | R:5’-CCACAGAAACAACATCGATTTCTT -3’ |
| Bcl-2 | F:5’-TCCAATCCTGTGCTGCTA-3’ |
|  | R:5’-ACTCTGTGAATCCCGTTT-3’ |
| GAPDH | F:5’-CGGAGTCAACGGATTTGGTCGTAT-3’ |
|  | R:5’-AGCCTTCTCCATGGTGGTGAAGAC -3’ |
|  |  |
