# Supplementary material for: Nestin overexpression in hepatocellular carcinoma associates with epithelial-mesenchymal transition and chemoresistance
Source: J Exp Clin Cancer Res. 2016 Jul 13;35:111. doi: 10.1186/s13046-016-0387-y (PMC4944516; doi:10.1186/s13046-016-0387-y)
Supplement: Additional file 2: Table S2. — IC50s (μM/L) of anticancer drugs for HCC cells. (DOCX 14 kb) [file 13046_2016_387_MOESM2_ESM.docx]

**Supplementary Table 2：IC50s (μM/L) of anticancer drugs for HCC cells**

|  | Bel-7402 | Bel-7402/5-FU | Bel-7402/ADM | *p* value |
| --- | --- | --- | --- | --- |
| 5-FU | 31.84±2.261 | 329.22±8.889 | 121.81±3.454 | 0.004 |
| L-OHP | 18.32±1.567 | 35.88±1.223 | 27.33±2.098 | 0.002 |
| ADM | 2.44±0.123 | 2.87±0.341 | 3.56±0.542 | 0.004 |

NOTE: Data are mean ± SD of three independent experiments.
