# Supplementary material for: Nestin overexpression in hepatocellular carcinoma associates with epithelial-mesenchymal transition and chemoresistance
Source: J Exp Clin Cancer Res. 2016 Jul 13;35:111. doi: 10.1186/s13046-016-0387-y (PMC4944516; doi:10.1186/s13046-016-0387-y)
Supplement: Additional file 4: Figure S1. — The mRNA expression levels of EMT markers in different groups of Bel-7402 cells measured by quantitative real-time RT-PCR. (DOCX 13 kb) [file 13046_2016_387_MOESM4_ESM.docx]

**Supplementary Table 3：IC50s (****μM/L) of anticancer drugs for HCC cells**

|  |  | Bel-7402/5-FU | |  |  | Bel-7402/ADM |  |
| --- | --- | --- | --- | --- | --- | --- | --- |
|  | Control | si-Nestin | *p* Value |  | Control | si-Nestin | *p* Value |
| 5-FU | 320±8.927 | 47.97±1.152 | <0.05 |  | 150.08±3.427 | 27.46±1.466 | <0.05 |
| L-OHP | 36.09±1.617 | 24.71±0.919 | <0.05 |  | 27.02±0.837 | 18.83±0.527 | <0.05 |
| ADM | 2.99±0.127 | 1.86±0.027 | <0.05 |  | 3.26±0.150 | 2.7±0.017 | <0.05 |

NOTE: Data are mean ± SD of three independent experiments.
